# Impact of baseline and nadir neutrophil index in non-small cell lung cancer and ovarian cancer patients: Assessment of chemotherapy for resolution of unfavourable neutrophilia

**DOI:** 10.1186/1479-5876-11-189

**Published:** 2013-08-15

**Authors:** Andreas Carus, Howard Gurney, Val Gebski, Paul Harnett, Rina Hui, Richard Kefford, Nicholas Wilcken, Morten Ladekarl, Hans von der Maase, Frede Donskov

**Affiliations:** 1 Department of Oncology and department of experimental clinical oncology, Aarhus University Hospital, Noerrebrogade 44, Bldg. 5, DK-8000, Aarhus, Denmark; 2 Department of Medical Oncology, Crown Princess Mary Cancer Centre, Westmead Hospital, Sydney, Australia; 3 NHMRC Clinical Trials Centre, University of Sydney, Sydney, Australia; 4 Department of Oncology, Copenhagen University Hospital, Copenhagen, Denmark

**Keywords:** Lung cancer, Ovarian cancer, Neutrophils, Tumour microenvironment, Prognostic factor

## Abstract

**Background:**

Chronic inflammation has been recognized to foster tumour development. Whether chemotherapy can be used to neutralize chronic inflammation is unclear.

**Methods:**

We evaluated baseline and nadir neutrophils in 111 patients (pts.) with non-small cell lung cancer (NSCLC) and 118 pts. with ovarian cancer (OC) treated with chemotherapy administered with dose-individualization to achieve nadir neutropenia of 1.5. We used predefined baseline neutrophil cut-offs 4.5 × 10^9^/L (NSCLC) and 3.9 × 10^9^/L (OC).

**Results:**

Absence of chemotherapy-induced nadir neutropenia (CTCAE grade 0, neutrophils ≥ LLN) was seen in 23% of OC and 25% of NSCLC pts. Absence of nadir neutropenia was associated with decreased overall survival (OS) compared with presence (>grade 0) of neutropenia (9 vs. 14 months, *P* = 0.004 for NSCLC and 23 vs. 56 months; *P* = 0.01 for OC). Obtaining grade 3/4 neutropenia did not improve survival compared with grade 1/2 neutropenia. In multivariate analyses, baseline neutrophils ≥4.5 × 10^9^/L (HR: 2.0; 95% CI: 1.11-3.44;P = 0.02) and absence of nadir neutropenia (HR: 1.6; 95% CI: 1.02-2.65;P = 0.04) for NSCLC and absence of nadir neutropenia (HR: 1.7; 95% CI: 1.04;2.93;P = 0.04) for OC were independently associated with short OS.

Three prognostic neutrophil index (NI) groups were defined. Favourable NI: low baseline neutrophils and presence of nadir neutropenia (>grade 0), Intermediate NI: elevated baseline neutrophils and presence of nadir neutropenia (>grade 0), and Poor NI: elevated baseline neutrophils and absence of nadir neutropenia (grade 0). For NSCLC patients, the median OS was 18.0, 13.4, and 8.8 months for favourable, intermediate and poor NI, respectively (fav vs. poor *P* = 0.002; fav vs. intermed *P* = 0.04; and intermed vs. poor *P* = 0.03). For OC patients, median OS was 69, 52 and 23 months for favourable, intermediate and poor NI, respectively (fav vs. poor *P* = 0.03; fav vs. intermed *P* = 0.3; and intermed vs. poor *P* = 0.02). Interestingly, survival rates in the intermediate NI groups indicated that individualised dose of chemotherapy to induce neutropenia may partly overcome the negative impact of elevated baseline neutrophils.

**Conclusions:**

A neutrophil index comprising elevated baseline neutrophils and absence of neutropenia identified a high risk group of NSCLC and ovarian cancer patients with only modest effect of chemotherapy. New treatment options for this subset of patients are required.

## Background

Although cancer begins as a single cell and initially develops as a clone, by the time a tumour is clinically detectable, it is no longer a mass of isolated, identical, neoplastic cells [1]. It has been realized that tumours are composed of an assemblage of cell types that communicate and collaborate, including cancer cells, cancer stem cells, endothelial cells, pericytes, fibroblasts and tumour-promoting inflammatory cells [2]. Thus, multiple non-malignant cell types are recruited to become components of the tumour and contribute to the hallmarks of cancer [3].

Among inflammatory cells, the realisation of the negative effect of neutrophils has recently begun to emerge [4]. Several studies have demonstrated that tumours stimulate neutrophils to promote angiogenesis and immunosuppression, as well as migration, invasion, and metastasis [5]. In clinical trials, the prognostic role of tumour-infiltrating neutrophils, elevated blood neutrophils, and elevated blood neutrophil/lymphocyte ratio has been clearly associated with poor clinical outcome in several human cancers, most notably in renal cell carcinoma, melanoma, colorectal cancer, hepatocellular carcinoma, cholangiocarcinoma, glioblastoma, GIST, gastric, esophageal, lung, ovarian, head and neck, and cervical cancer [6-8]. A striking finding is the notion that high baseline neutrophil count hinder benefit from surgery, chemoradiotherapy, radiofrequency ablation, and chemotherapy [6]. Consequently, neutrophils, in addition to tumour cells, are potential targets for cancer therapy [9,10].

Traditionally, neutropenia in relation to chemotherapy has been regarded as a harmful side effect that should be avoided. However, several retrospective studies have suggested an inferior outcome for patients failing to achieve mild neutropenia during chemotherapy for breast, ovarian, and non-small cell lung cancers (NSCLC) as well as Hodgkin’s lymphoma [11-21] and for targeted therapy with sunitinib, cetuximab and imatinib [22-25]. Nevertheless, it is unclear whether baseline and nadir neutrophils are linked in the individual patient.

In the present study, we evaluated the prognostic impact of combined baseline and nadir neutrophils in an institution with a standard practise of individualizing chemotherapy dosing upwards or downwards to achieve target nadir neutropenia of 1.5 × 10^9^/L [26,27]. We chose patients with NSCLC and ovarian cancer (OC) as predefined baseline neutrophil cutoff values of 4.5 × 10^9^/L and 3.9 × 10^9^/L, respectively, have been determined from previous studies [28,29]. We identified a new prognostic neutrophil index by combining baseline and nadir neutrophil values in patients with NSCLC and ovarian cancer.

## Methods

### Patient population

Data from patients diagnosed with non-small cell lung cancer (stage III-IV) and advanced ovarian cancer (stage I-IV) treated with chemotherapy between 1997 and 2005 were collected from patient records at the Department of Medical Oncology at Crown Princess Mary Cancer Centre Westmead in Sydney Australia.

Eligibility criteria were a complete medical record within three cycles of chemotherapy and a full set of baseline and nadir laboratory data. All available medical files were reviewed and a number of files were excluded due to lack of essential data. Patients routinely had a nadir blood count measured 10 to 17 days after chemotherapy or as appropriate according to schedule. The highest grade of myelosuppression at nadir was recorded. Demographics, type of chemotherapy, use of G-CSF, clinical, laboratory, and survival data were collected. Stage was graded according to TNM 2002 or FIGO 1998 as appropriate. No upfront G-CSF was used. Toxicity and laboratory data were graded according to CTCAE v.3.0. Survival data were updated May 2010.

The study has received institutional review board and Ethics Committee approval. (Sydney West Area Health Service Human Research Ethics Comitee). Since this was a non-interventional, retrospective, subject-anonymized study, written patient consent was not required by the ethics committee.

### Treatment and toxicity-adjusted dose modification

A protocol of chemotherapy-toxicity adjusted dosing (CTAD) was implemented as clinical standard practice in the mid-1990s at the institution [26]. After an initial administration of chemotherapy based on standard body surface area, subsequent doses were adjusted upwards or downwards in each patient to yield target nadir neutrophil count of 1.5 × 10^9^/L (i.e. CTCAE grade 1 neutropenia). If target neutropenia was not reached and other haematological or non-haematological toxicity was ≤ Grade 2, the subsequent dose of chemotherapy was *increased* by 15-20%. In contrast, if the patient experienced significant non-haematological toxicity > Grade 2 toxicity or severe neutropenia, the dose was *reduced* by 15-20%. Otherwise the dose remained unchanged [26].

### Statistical analysis

Summary statistics were performed to estimate relevant baseline patient demographic and disease characteristics. Relative chemotherapy intensity was calculated as the actual cumulated dose of chemotherapy divided by the standardized cumulated dose according to expected number of chemotherapy cycles. The impact on outcome was explored for all patients in each tumour type as well as in the subgroups of patients receiving the most frequent chemotherapy regimen.

Based on previous studies identifying baseline neutrophil count as independent prognostic factors in NSCLC [28] and ovarian cancer [29] baseline neutrophil counts were dichotomized according to the pre-defined cutoff values of 4.5 × 10^9/L for NSCLC and 3.9 × 10^9/L for ovarian cancer. Patients who developed various CTCAE version 3 grades of myelosuppression were compared with those who did not. The relationship between assessed parameters and overall survival (OS) was evaluated using the method of Kaplan–Meier and log-rank tests. Multivariate Cox regression models were constructed to report hazard ratios (HRs) for OS. Factors with *P <* 0.1 in univariate analysis were dichotomized, except for age which was treated as a continuous variable, and included in the multivariate model.

For NSCLC patients age, performance status, tumour stage, presence of bone metastases, baseline haemoglobin level, baseline neutrophil count above the predefined cut-off, nadir neutropenia grade ≥0, and relative chemotherapy intensity were included in the multivariate analysis.

For ovarian cancer patients age, performance status, FIGO stage, presence of ascites, residual disease, normalisation of CA125, baseline neutrophil count above the predefined cut-off, and nadir neutropenia grade ≥0 were included in the multivariate analysis.

Differences in baseline neutrophil count in different tumour stages was compared with either the nonparametric Mann–Whitney test (NSCLC patients) or Kruskal-Wallis test (ovarian cancer patients).

All reported *P*-values were two-tailed; an alpha-value below 0.05 was considered statistically significant. The analyses were performed using SPSS version 20.0.0 (IBM Corporation, NY, USA).

## Results

A total of 111 patients with non-small cell lung cancer (NSCLC) and 118 patients with ovarian cancer (OC) fulfilled inclusion criteria for the present study. Patient characteristics are shown in Tables 1 and 2. For NSCLC patients, 71 (64%) achieved ≥ grade 2 nadir neutropenia (i.e., neutrophils <1.5 × 10^9^/L), 15 (14%) achieved grade 1 nadir neutropenia (i.e., neutrophils < LLN to 1.5 x 10^9^/L), and 25 (23%) had no chemotherapy-induced neutropenia (grade 0, i.e., neutrophils ≥ LLN). For ovarian cancer patients 64 patients (54%) achieved ≥ grade 2 neutropenia, 24 (20%) achieved grade 1 neutropenia, and 30 (25%) had no neutropenia (grade 0). One ovarian cancer patient received G-CSF due to neutropenia. Dose increase due to absence of target neutropenia occurred in 19% of NSCLC and 23% of OC patients. Dose reduction due to haematological or non-haematological toxicity occurred in 36% of NSCLC patients and 25% of OC patients.

**Table 1:** Patient characteristics for NSCLC patients (N = 111)

**Characteristic**	**N**	**%**
Median age, years	61	
Age range, years	32–84	
Sex		
Male	53	48
Female	58	52
NSCLC stage		
IIIa	23	21
IIIb	36	32
IV	52	47
ECOG performance		
0	51	46
1	52	47
2	7	6
Missing	1	1
Baseline neutrophils ≥ 4.5		
Yes	90	81
No	21	19
Tumour Histology		
Adenocarcinoma	58	52
All other	53	48
Bone metastases		
Yes	17	15
No	90	81
Missing	4	4
Chemotherapy regimens		
Carboplatin based	87	78
Cisplatin based	4	4
Other	20	18

Abbreviations: *NSCLC* non-small-cell lung cancer, *ECOG* Eastern Cooperative Oncology Group.

**Table 2:** Patient characteristics for ovarian cancer patients (N = 118)

**Characteristic**	**N**	**%**
Median age, years	58	
Age range, years	33–82	
FIGO stage		
1	16	14
2	17	14
3	71	60
4	14	12
ECOG performance		
0	70	59
1	41	35
2+	6	5
Missing	1	1
Baseline neutrophils > 3.9		
Yes	98	83
No	20	17
Optimal debulking		
No	36	31
Yes	80	68
Missing	2	<2
Ascites at surgery		
No	32	27
yes	79	67
Missing	7	6
Normalized CA125		
No	16	14
yes	96	81
Missing	6	5
Chemotherapy regimens		
Carbo + Tax	73	62
Carbo monotherapy	33	28
Carbo + other	12	10

Abbreviations: *FIGO* International Federation of Gynaecology and Obstetrics, *ECOG* Eastern Cooperative Oncology Group; *CA125* Cancer Antigen 125; *Carbo* Carboplatin; *Tax* Taxol (Paclitaxel).

### Impact of baseline and nadir neutrophils in univariate analyses

#### ***Non-small cell lung cancer***

**Figure 1:**
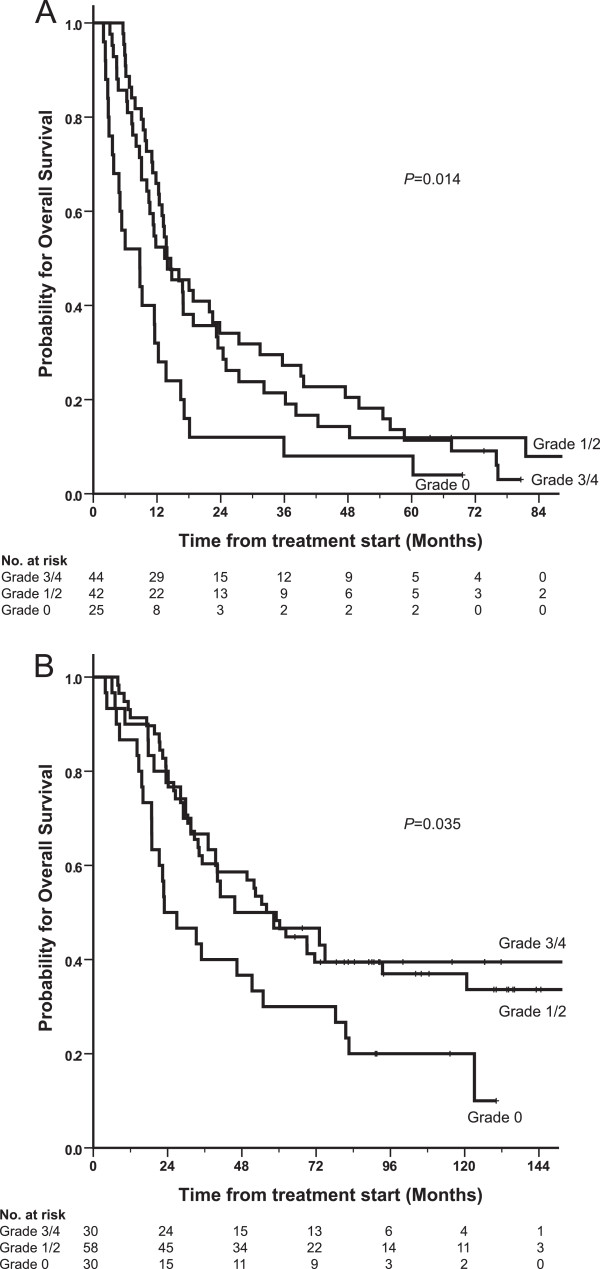
**Kaplan-Meier plot of overall survival for NSCLC patients (A) (N = 111) and ovarian cancer patients (B) (N = 118) stratified for neutropenia grade at nadir.** Grade 0, neutrophils ≥ LLN; grade 1, neutrophils < LLN to 1.5 × 10^9^/L; grade 2, <1.5 - 1.0 × 10^9^/L; grade 3, <1.0 – 0.5 × 10^9^/L; grade 4, < 0.5 × 10^9^/L. P-values obtained from log-rank test.

Overall median survival was 13.0 months for NSCLC patients with a median follow-up of 6.7 years. Patients failing to achieve any grade of nadir neutropenia (i.e., neutrophils ≥ LLN) after chemotherapy had decreased survival rate compared with patients who obtained any grade of neutropenia (i.e. >grade 0 neutropenia), median survival 9 vs. 14 months; (*P* = 0.004). Obtaining grade 3 or 4 neutropenia did not improve survival rate compared with grade 1 or 2 neutropenia (Figure 1A).

Patients with baseline elevated blood neutrophils above or equal to the predefined cutoff of 4.5 × 10^9^/L had decreased OS compared with patients with baseline neutrophil counts below 4.5, median survival 11.6 vs. 18.0 months; (*P* = 0.02). The impact of relative dose intensity of chemotherapy was significantly associated with overall survival (*P* = 0.003)*.* Other factors associated with poor overall survival were performance status >0 (*P* = 0.08), presence of bone metastases (*P* = 0.003), Stage IV cancer (*P* = 0.001), and haemoglobin count below lower limit of normal (*P* = 0.02).

We observed a non-significant trend towards higher median values of baseline neutrophil counts for NSCLC patients with increasing stage (IIIa vs. IIIB vs. IV; median 5.8, 5.5, and 6.5 × 10^9^/L, respectively; P = 0.13).

#### ***Ovarian cancer***

Median survival was 50 months for all ovarian cancer patients with a median follow-up time of 9.0 years. Patients who failed to achieve neutropenia (i.e., neutrophils ≥ LLN, grade 0 neutropenia) had less than half the median survival compared to patients achieving any grade of neutropenia (23 vs. 56 months; *P* = 0.01). Obtaining grade 3 or 4 neutropenia did not improve survival rate compared with grade 1 or 2 neutropenia (Figure 1B). We observed no statistically significant survival impact of baseline elevated neutrophils above or below the predefined cutoff of 3.9x10^9^/L (*P* = 0.3). Relative chemotherapy dose did not impact overall survival (HR 1.00; 95% CI 0.98–1.03; *P* = 0.6). Other factors significantly associated with poor overall survival were performance status >0 (*P* = 0.001), residual disease >1 cm (*P* = 0.0001), presence of ascites (*P* = 0.0001), and failure to normalize serum CA125 (*P* = 0.003).

We observed a non-significant trend towards higher baseline neutrophil counts with increasing FIGO stage (I, II, III, and IV; median 4.8, 5.3, 5.9, and 7.2 × 10^9^/L, respectively; P = 0.08).

### Multivariate analyses

For NSCLC patients factors independently associated with short survival in multivariate analysis were failure to achieve neutropenia with chemotherapy (i.e., grade 0 neutropenia), baseline neutrophil count above or equal to 4.5 × 10^9^/L, relative chemotherapy intensity <100%, and TNM stage IV (Table 3).

**Table 3:** Multivariate analysis of association between on-treatment neutropenia and OS for non-small cell lung cancer and ovarian cancer

**Tumour type**	**HR for death (95% CI)**	**P-value**
**NSCLC**		
Neutropenia grade 0	1.6 (1.02;2.65)	0.04
Baseline neutrophils ≥ 4.5	2.0 (1.11;3.44)	0.02
Stage IV cancer	1.8 (1.18;2.71)	0.006
Relative chemo intensity < 100%	1.7 (1.11;2.60)	0.01
**Ovarian cancer**		
Neutropenia grade 0	1.7 (1.04;2.93)	0.04
Residual disease > 1 cm	2.9 (1.73;4.81)	0.0001
Age at diagnosis	1.02 (1.00;1.05)	0.04
Ascites present	2.2 (1.11;4.23)	0.02
CA125 not normalized	3.2 (1.75;6.00)	0.0001

Factors with multivariate significance level <0.05 are shown.

Abbreviations: *OS* overall survival; *NSCLC* Non-Small Cell Lung Cancer; *Grade 0* neutropenia at nadir ≥ 2.0 × 10^9/L; *CA125* Cancer antigen 125.

For ovarian cancer patients the following factors were independently associated with short survival in multivariate analysis: failure to achieve neutropenia with chemotherapy (i.e., grade 0 neutropenia), minimal residual disease >1 cm, increasing age, presence of ascites, and failure to normalize CA125 after chemotherapy (Table 3).

### Impact of baseline and nadir neutrophil index

**Figure 2:**
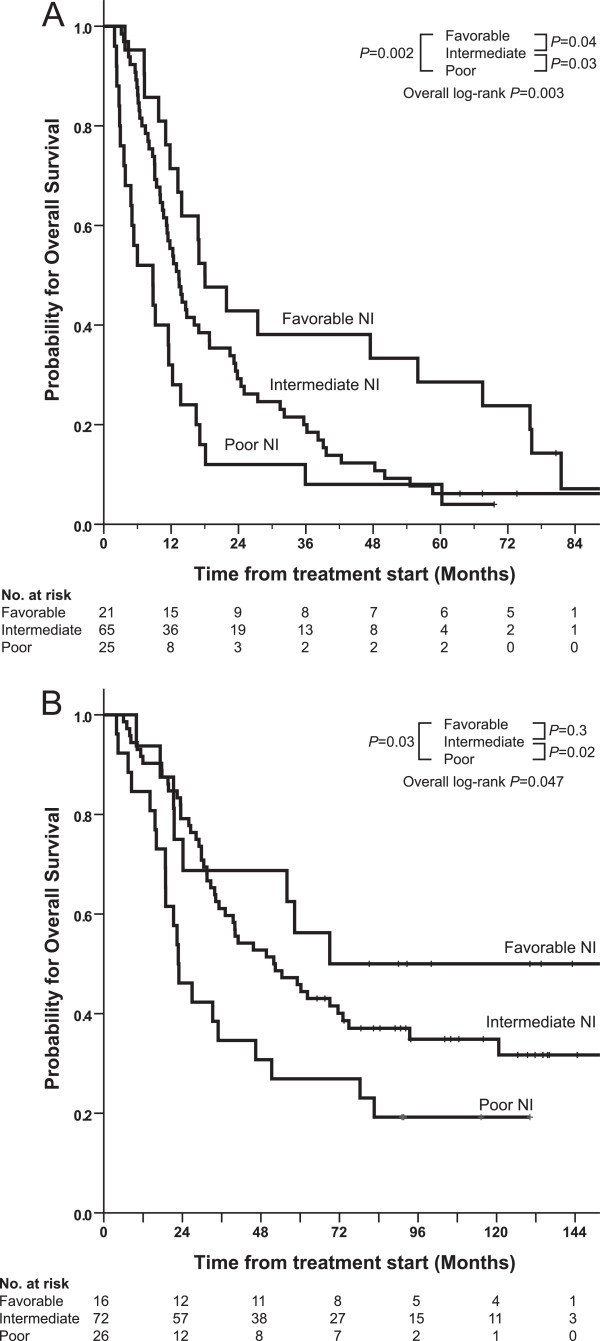
**Kaplan-Meier plot of overall survival for NSCLC patients (A) (N = 111) and ovarian cancer patients (B) (N = 118) stratified for *favourable neutrophil index (NI) *(low baseline neutrophils and presence of nadir neutropenia), *intermediate neutrophil index *(elevated baseline neutrophils and presence of nadir neutropenia), and *poor neutrophil index *(elevated baseline neutrophils and absence of nadir neutropenia) prognostic group.** A fourth group with low baseline neutrophils and absence of nadir neutropenia comprised only 4 patients with ovarian cancer and no NSCLC, and was not classified.

To evaluate the combined prognostic impact of both baseline and nadir neutrophils we performed a four-group analyses. Based on predefined baseline neutrophil cutoff values and nadir neutropenia grade (0 *vs.* >0), we identified a favourable neutrophil index prognostic group (comprising patients with low baseline neutrophils and presence (>grade 0) of nadir neutropenia), an intermediate neutrophil index prognostic group (comprising patients with elevated baseline neutrophils and presence (>grade 0) of nadir neutropenia), and a poor neutrophil index prognostic group (comprising patients with elevated baseline neutrophils and absence of (grade 0) nadir neutropenia). The fourth potential group of low baseline neutrophils and grade 0 nadir neutropenia comprised only 4 patients with ovarian cancer and no NSCLC patients, and was therefore not classified. For NSCLC patients, the median OS was 18.0, 13.4, and 8.8 months for favourable, intermediate and poor neutrophil index prognostic group, respectively (Figure 2A): (favourable vs. poor *P* = 0.002, favourable vs. intermediate *P* = 0.04, intermediate vs. poor *P* = 0.03). Number of dose increase and dose reduction in the intermediate group was not statistically different from the poor prognostic group. For ovarian cancer patients, median OS was 69, 52 and 23 months for favourable, intermediate and poor neutrophil index prognostic group, respectively (Figure 2B): favourable vs. poor *P* = 0.03, favourable vs. intermediate *P* = 0.3, intermediate vs. poor *P* = 0.02. A significantly higher number of patients in the intermediate group had dose reduction compared with the poor prognostic group (*P* = 0.017) whereas no difference in dose increase or relative chemotherapy dose intensity was observed between the intermediate and poor prognostic group.

## Discussion

To our knowledge, this is the first study to identify a prognostic neutrophil index in non-small cell lung cancer (NSCLC) and ovarian cancer patients taking into account both pre-treatment and post-treatment neutrophils. Using baseline and nadir neutrophils in a combined prognostic index we were able to identify a subgroup of patients - with baseline neutrophils above the pre-defined cutoffs and failure to achieve neutropenia following chemotherapy - who had a dismal prognosis comprising approximately one quarter of the patient population. In this poor neutrophil index group it appears that chemotherapy had minimal impact for resolution or neutralization of the negative effect of neutrophils despite the use of a protocol designed to induce neutropenia. It is unknown whether further dose escalation in those individuals would have had a positive benefit. It might be that the effect of chemotherapy in these patients has reached its ceiling and other means of therapy are required [3]. In contrast, patients with baseline neutrophils below the pre-defined cutoff and who obtained neutropenia below lover limit of normal (>grade 0) did benefit from chemotherapy and had a two to three-fold better overall survival.

High baseline neutrophil count hinder benefit from surgery, chemoradiotherapy, radiofrequency ablation, and chemotherapy in several human cancers (reviewed in [6]). Our findings validate the cutoff baseline neutrophil count above or equal to 4.5 × 10^9^/L in NSCLC patients previously identified by Teramukai *et al.*[28] as an independent prognostic factor for poor outcome. Other studies in NSCLC have also demonstrated an adverse prognostic effect of high baseline neutrophil count [30-34]. Data from the Teramukai study also suggested a link between high pre-treatment neutrophil count and increased treatment-related non-haematological toxicity. The incidence of grade 3 or 4 non-haematological toxicity within the first three cycles of treatment was significantly higher in the high-neutrophil group compared to the low neutrophil group. Moreover, none of the patients in the high-neutrophil group who experienced grade 3 or 4 non-haematological toxicity within the first three cycles completed the planned six cycles [28]. This data suggests that simply increasing the dose of chemotherapy to induce neutropenia may be difficult to achieve in those patients with a high neutrophil count. However, it should be noted that in the study by Teramukai, 47% of patients had elevated baseline neutrophil count above 4.5, whereas in our study we had 81% of patients with elevated baseline neutrophil count above 4.5. This means that we have a higher proportion of patients in our study with poor prognostic features. Nonetheless, we were able to demonstrate benefit from chemotherapy in a group of patients.

High neutrophil count has been shown to be negative prognositc factor for other tumour types. In colorectal cancer patients treated with anti-VEGF containing regimens, elevated neutrophil count predicted poor OS and RFS [35]. In gastrointestinal stromal tumours (GIST) elevated baseline neutrophil count correlated with initial as well as late resistance to imatinib treatment [36]. In metastatic renal cell carcinoma, elevated baseline blood neutrophil count has been integrated as a validated prognostic factor in the Heng criteria for patients treated with targeted therapy [37] and both elevated blood neutrophils as well as presence of intratumoural neutrophils were independently correlated with poor survival in patients treated with cytokines [38]. Taken together, elevated neutrophils have serious clinical implications. However, we were unable to confirm the cutoff baseline neutrophils count above 3.9 × 10^9^/L in ovarian cancer patients identified by Banerjee *et al.*[29], probable due to small sample size in our series.

Treatment-induced neutropenia was found to be an independent favourable variable in our population. Approximately one quarter of the patients failed to achieve any neutropenia despite the use of a chemotherapy toxicity adjusted protocol, where the goal was to induce neutropenia, and these patients had a worse survival. Moreover, we observed that severe neutropenia (i.e., grade 3–4) was no better than mild neutropenia (i.e., grade 1–2) but that both were better than no neutropenia (i.e., grade 0) in terms of improved median survival. In other words, it is the presence, but not the severity, of neutropenia that is prognostic. Similar to our results, Di Maio et al. [13] and Kishida et al. [15] have previously shown an inferior survival in NSCLC patients who experienced no treatment-related neutropenia (grade 0) but that no apparent additional benefit was seen with higher than grade 1 neutropenia. If validated prospectively, these findings may impact future routine clinical practice.

The novel, important finding from our data, that may have practical implications, is that among patients with higher baseline neutrophils, approximately 75% obtained neutropenia > grade 0 following chemotherapy and subsequently had a significantly improved overall survival compared to those 25% who experienced no neutropenia. These figures were almost identical in NSCLC and OC patients. Similar assessments have been done in colorectal cancer using neutrophil/lymphocyte ratio (NLR) as a marker [39]. Baseline blood NLR (>5) was shown to independently predict poor OS. Importantly, normalization of the NLR after one cycle of chemotherapy was observed in a subset of patients, which resulted in a 2-month PFS improvement (5.8 vs. 3.7 months) compared with patients without NLR normalization [39]. However, normalization of the NLR after one cycle of chemotherapy did not result in a statistically significant improvement in OS compared with patients without NLR normalization. No chemotherapy dose individualization was done in these patients based on toxicity or nadir neutrophil count, as was done in our study. Thus, these data imply that not all patients with elevated baseline neutrophil are “protected from the benefits of chemotherapy” as suggested by Maione et al. [40]. In our series both the higher baseline count and absence of treatment-induced neutropenia were independent adverse prognostic factors and thus were not linked. Moreover, the relative dose intensity of chemotherapy was an independent prognostic factor in NSCLC. This implies that the dose of chemotherapy matters and may partially overcome the negative effects of an elevated baseline neutrophil count. The results in our intermediate prognosis group patients in both cancer groups seem to support this concept. Patients with a relatively high baseline neutrophil count who developed neutropenia from chemotherapy (i.e., the intermediate prognostic group) had a statistically significantly better survival than those patients who did not achieve treatment-induced neutropenia (i.e., the poor prognostic group). However, survival was still lower compared to patients with relatively low neutrophil count at baseline (i.e., the favorable group).

Inflammatory cells, including neutrophils, influence many aspects of cancer initiation, progression and metastatic potential in the tumour microenvironment [41]. Recruitment of neutrophils from the bone marrow to sites of inflammation is a well-documented process guided by chemochine-, lipid-, complement- and N-formylated peptide chemoattractant mediators [5]. However, human studies evaluating at the same point in time peripheral blood inflammatory cells and intratumoural inflammatory cells are scarce. Recently, in resected stage I-IIIA NSCLC patients [8] we demonstrated densities of tumour-associated CD66+ neutrophils and CD163+ macrophages were correlated with adverse clinical prognostic factors as well as CRP and white blood cell (WBC) systemic inflammatory markers. We also demonstrated that elevated blood CRP and WBC were associated with short recurrence free interval (RFS) and overall survival but tumour-associated neutrophils and macrophages were not directly correlated with RFS or overall survival [8]. In patients with metastatic renal cell carcinoma, elevated blood neutrophils as well as presence of CD66b+ intratumoural neutrophils were independently correlated with poor OS in multivariate analysis [38]. This suggests that the tumour microenvironment may have two compartments, a local and a systemic and that both compartments may be important targets for therapy. Assessment of chemotherapy for resolution of chronic inflammation is a new paradigm and should be evaluated further in randomized trials incorporating the neutrophil index in the study design. Targeting chronic inflammation in the tumour microenvironment is an area of intense research. In Table 4 we have outlined drugs approved or in clinical trials that target inflammatory immune cells.

**Table 4:** Drugs targeting inflammatory immune cells in the tumour microenvironment of solid tumours (excluding haematological cancers)

**CT identifier**	**Drug**	**Method of action**	**Cancer**	**Approved/phase study**
**Targeting T lymphocytes**				
NCT01844505	Nivolumab	Anti-PD1	mMM	Phase 3
NCT01642004	Nivolumab	Anti-PD1	NSCLC	Phase 3
NCT01354431	Nivolumab	Anti-PD1	mRCC	Phase 2
NCT00094653	Ipilimumab	Anti-CTLA4	mMM	Approved
NCT00861614	Ipilimumab	Anti-CTLA4	PC	Phase 3
NCT01450761	Ipilimumab	Anti-CTLA4	SCLC	Phase 3
NCT01285609	Ipilimumab	Anti-CTLA4	NSCLC	Phase 3
NCT01693783	Ipilimumab	Anti-CTLA4	Cervical cancer	Phase 2
NCT01860430	Ipilimumab	Anti-CTLA4	HNSCC	Phase 1B
NCT00257205	CP-675,206	Anti-CTLA4	mMM	Phase 3
**Blocking immune cell recruitment**				
NCT01346358	IMC-CS4	CSFR1 antagonist	Advanced tumours	Phase 1
NCT01316822	ARRY-382	CSFR1 antagonist	Advanced tumours	Phase 1
NCT01015560	MLN1202	Anti-CCR2	Bone metastases	Phase 1
NCT01032122	Rituximab	Anti-CD20	mMM	Phase 1
NCT01376713	Ofatumumab	Anti-CD20	mMM	Phase 2
NCT01456585	CP-870,893	Anti-CD40	mPC	Phase 2
NCT01103635	CP-870,893	Anti-CD40	mMM	Phase 1
NCT00607048	CP-870,893	Anti-CD40	Solid tumours	Phase 1
**Reprogramming immune cells**				
NCT00169104	G-CSF	Sustained neutrophilia	mBC	Phase 1/2
NCT00014456	G-CSF		Solid tumours	Phase 1
NCT00070629	Promune	TLR9 agonist	NSCLC	Phase 2
NCT00043394	Promune	TLR9 agonist	BC	Phase 2
NCT00070642	Promune	TLR9 agonist	MM	Phase 2
NCT00043407	Promune	TLR9 agonist	RCC	Phase 1/2
NCT00292045	Promune	TLR9 agonist	PC	Phase 1
NCT00669292	Promune	TLR9 agonist	Esophageal cancer	Phase 1/2
Multiple	Vaccines (Multiple targets)	T-cell activation	Multiple cancers	
**TH2-- > TH1 reprogramming**				
NCT01642290	Anti-OX40	Anti-OX40	mBC	Phase 1/2
NCT01644968	Anti-OX40	Anti-OX40	Advanced cancer	Phase 1
NCT01689870	Anti-OX40	Anti-OX40	mMM	Phase 1/2

For an indepth review of the current landscape of targeting chronic inflammation, see Coussens [9]. CT identifier is to clinicaltrials.gov.

Abbreviations: *mMM* metastatic malignant melanoma, *NSCLC* non-small cell lung cancer, *mRCC* metastatic renal cell carcinoma, *PC* prostate cancer, *SCLC* small-cell lung cancer, *HNSCC* head-and-neck squamous cell cancer, *mBC* metastatic breast cancer.

Further research in the area of chronic inflammation and cancer is encouraged.

Limitations of our study are the low sample size, the retrospective design, inhomogeneous chemotherapy regimens, and patient accrual over a long period of time. Additionally, the requirement for data for three cycles of treatment excludes patients who died early, potentially skewing the survival analysis. However, the present study was conducted in two independent, different tumour types with achievement of almost identical results.

## Conclusions

In conclusion, absence of chemotherapy-induced neutropenia was an independent adverse prognostic factor in NSCLC and ovarian cancer patients. By combining baseline elevated neutrophil count and absence of neutropenia, we identified a poor prognostic group who appeared to have little benefit from chemotherapy despite a dose escalation protocol. New treatment options for this subset of patients are required. Importantly, we found an intermediate prognostic group where the induction of neutropenia by chemotherapy may have partially overcome the negative impact of elevated baseline neutrophils leading to a better survival. This has implications for dose individualisation in this subgroup. The combined prognostic neutrophil index comprising both baseline and nadir neutrophil count is a potentially new and important finding that requires validation in larger, prospective studies.

## Competing interests

The authors declare that they have no competing interests.

## Authors’ contributions

FD collected patient data and planned the study with AC, HG and HvdM. AC and FD made an equal contribution in data analysis, manuscript planning, and writing. HG participated in manuscript writing and data analysis. VG provided statistical analysis and advice. PH, RH, HG, RK and NW provided patients and revised the manuscript critically. All authors read and approved the final manuscript.
